# Identification of potential CepR regulated genes using a *cep *box motif-based search of the *Burkholderia cenocepacia *genome

**DOI:** 10.1186/1471-2180-6-104

**Published:** 2006-12-22

**Authors:** Catherine E Chambers, Erika I Lutter, Michelle B Visser, Peggy PY Law, Pamela A Sokol

**Affiliations:** 1 Department of Microbiology and Infectious Diseases, University of Calgary Health Sciences Center, Calgary, Alberta, Canada

## Abstract

**Background:**

The *Burkholderia cenocepacia *CepIR quorum sensing system has been shown to positively and negatively regulate genes involved in siderophore production, protease expression, motility, biofilm formation and virulence. In this study, two approaches were used to identify genes regulated by the CepIR quorum sensing system. Transposon mutagenesis was used to create *lacZ *promoter fusions in a *cepI *mutant that were screened for differential expression in the presence of *N*-acylhomoserine lactones. A bioinformatics approach was used to screen the *B. cenocepacia *J2315 genome for CepR binding site motifs.

**Results:**

Four positively regulated and two negatively regulated genes were identified by transposon mutagenesis including genes potentially involved in iron transport and virulence. The promoter regions of selected CepR regulated genes and site directed mutagenesis of the *cepI *promoter were used to predict a consensus *cep *box sequence for CepR binding. The first-generation consensus sequence for the *cep *box was used to identify putative *cep *boxes in the genome sequence. Eight potential CepR regulated genes were chosen and the expression of their promoters analyzed. Six of the eight were shown to be regulated by CepR. A second generation motif was created from the promoters of these six genes in combination with the promoters of *cepI*, *zmpA*, and two of the CepR regulated genes identified by transposon mutagenesis. A search of the *B. cenocepacia *J2315 genome with the new motif identified 55 *cep *boxes in 65 promoter regions that may be regulated by CepR.

**Conclusion:**

Using transposon mutagenesis and bioinformatics expression of twelve new genes have been determined to be regulated by the CepIR quorum sensing system. A *cep *box consensus sequence has been developed based on the predicted *cep *boxes of ten CepR regulated genes. This consensus *cep *box has led to the identification of over 50 new genes potentially regulated by the CepIR quorum sensing system.

## Background

*Burkholderia cenocepacia*, belongs to a group of nine related species with common phenotypes, but distinct genotypes collectively named the "*Burkholderia cepacia *complex" (Bcc) [1,2]. The Bcc are opportunistic pathogens in immunocompromised and cystic fibrosis (CF) patients but have also been isolated from plant rhizopheres as well as urban and suburban soils [1-3].

The ability of bacteria to adapt to diverse environments is dependent on the coordinate regulation of factors required to survive and proliferate in each niche. The CepIR quorum sensing system is one regulatory network that contributes to the response of *B. cenocepacia *to environmental signals (reviewed in [4,5]). Quorum sensing allows bacterial populations to coordinate gene expression in response to population density. CepIR belongs to a group of more than 50 quorum sensing systems that are homologous to the LuxIR system of *Vibrio fishceri *[6,7]. LuxI homologs are *N*-acyl homoserine lactone (AHL) synthases that generate AHL signal molecules that are released into the environment. LuxR homologs are transcriptional regulators that complex with AHL and typically bind to a *lux*-box overlapping the -35 sequence of a promoter to regulate transcription. The *lux*-box consensus sequence recognized by LuxR homologs typically consists of an inverted repeat with significant consensus among quorum sensing systems [6,8-10].

The CepIR system was originally identified in *B. cenocepacia *(formerly *B. cepacia*) K56-2 [11] and has subsequently been shown to be widely distributed throughout the Bcc [12,13]. CepI directs the synthesis of *N*-octanoyl homoserine lactone (OHL) and *N*-hexanoyl homoserine lactone (HHL) and *cepR *encodes for the transcriptional regulator [11-14]. CepR has been shown to negatively regulate its own expression, but positively regulate *cepI *expression at the transcriptional level [14]. The *cepIR *genes are involved in the regulation of the *pvdA *gene required for ornibactin biosynthesis [14], the *zmpA *and *zmpB *extracellular metalloproteases [15,16], the *aidA *gene involved in virulence in *Caenorhabditis elegans *[17-20], swarming motility and in at least some systems a functional CepIR quorum sensing system is necessary for biofilm formation [21-23]. The CepIR system has been shown to contribute to virulence in both plant and animal models. In *B. cepacia *ATCC 25416 mutations in *cepI *and *cepR *attenuated maceration in the onion-rot model [24]. The contribution of CepIR to the severity of *B. cenocepacia *infections has been demonstrated in two different animal models, a chronic respiratory infection model in rats and an acute intranasal infection model in Cftr^*(-/-) *^mice [16]. CepIR have also been shown to be important for virulence in *C. elegans *[25].

Proteomics and promoter based approaches have been used to identify genes regulated by the CepIR quorum sensing system. Proteome analysis was used to compare the protein profiles of *B. cenocepacia *strain H111 and an H111 *cepI *mutant [19]. Differences in expression were observed for 55 out of 585 proteins and partial N-terminal amino acid sequences were determined for peptide fragments of 11 proteins including AidA, FimA, and SodB. A promoter trap approach was used to identify positively regulated OHL-CepR dependent promoters in *B. cepacia *ATCC 25416 [17]. A library of ATCC 25416 fragments cloned upstream of a promoterless *lacZ *gene in a vector that also contained *cepR *was screened in *E. coli *in the presence and absence of OHL. Twenty-eight clones with genes upregulated in the presence of OHL were identified. The genes belonged to several functional classes; however, the only overlap in genes identified between the two studies was *aidA *[17,19]. Mutagenesis with a transposon containing a promoterless *lacZ *reporter was used to identify seven genes positively regulated by the *cepIR *quorum sensing system in *B. cenocepacia *strain K56-2, including *cepI *and *aidA *[20].

Identification of genes directly and indirectly regulated by CepR is a key step to understanding this regulatory system and the regulatory hierarchies that mediate the adaptation *B. cenocepacia *to the diverse environments it encounters. The above approaches search for genes regulated under defined *in vitro *conditions and therefore may not identify genes induced only in specific environmental niches including the plant or animal host. Only the study by Aguilar *et al*. [17] attempted to identify genes that are regulated by the direct interaction of CepR at the promoter.

LuxR homologs have been shown to bind to specific sequences referred to as *lux *boxes or the boxes for the gene designation of the respective *luxR *homolog such as *tra *boxes in the case of recognition sequences for *Agrobacterium tumefaciens *TraR [26-28]. These sequences have dyad symmetries and generally overlap the -35 RNA polymerase binding site. Lewenza et al. demonstrated that CepR was required for the expression of *cepI *in *B. cenocepacia *[11,14] and identified a putative *lux*-box like sequence with imperfect repeats that overlapped the -35 region of the putative *cepI *promoter [11]. Weingart et al. [20] demonstrated that CepR directly bound to a DNA fragment that contained the *cepI *promoter using electrophoretic mobility shift assays. They also mapped the transcriptional start site of *cepI *and using DNAase I footprinting experiments localized the CepR binding site to a region that closely corresponded to the *cep *box predicted by Lewenza et al[11]. In the present study, we used a functional genomics approach to identify genes in the *B. cenocepacia *J2315 sequence with a *cep *box-like sequence in their promoters. We confirmed by site-directed mutagenesis the *cep *box sequence located upstream of the *cepI *gene that is necessary for *cepI *transcription. Using selected *B. cenocepacia *CepR regulated genes we predicted a consensus *cep *box motif sequence and used that motif to search the *B. cenocepacia *J2315 genome to identify promoters potentially regulated by CepR.

## Results

### Functional analysis of the CepR binding site

**Figure 1:**
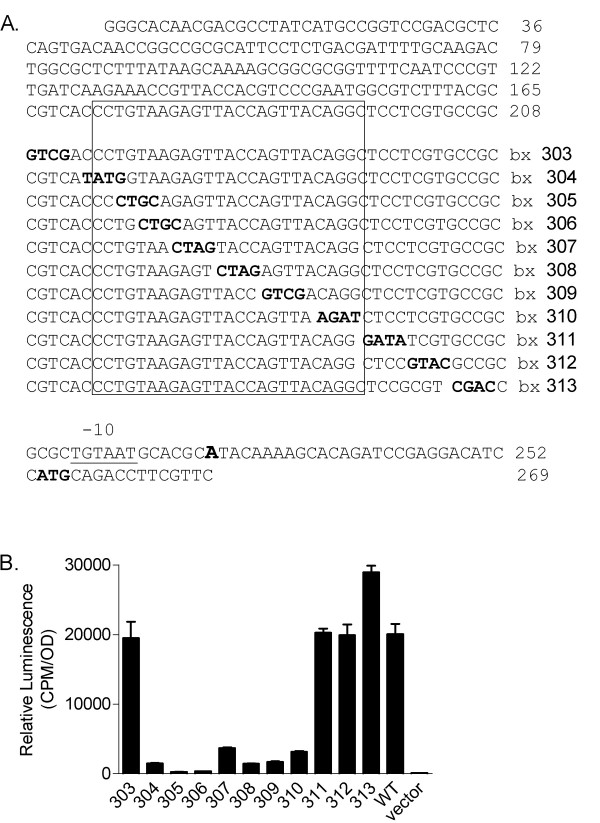
**Functional analysis of the CepR binding site**. A. Site directed mutagenesis was used to determine the effects of mutations on the luminescence activity of a *cepI::luxCDABE *fusion. The sequence upstream of the CepI ORF is shown. The ATG start codon is indicated by bold lettering and the predicted -10 hexamer is underlined. A series of 4 bp substitutions used to mutate the promoter region are indicated as bx303-313 and the *cep *box consensus sequence is enclosed in the rectangle. B. Expression of the *cepI::luxCDABE *fusions in *B. cenocepacia *K56-2. Luminescence (CPM) was measured at 22 hours and is represented as CPM/O.D. The numbers on the x axis indicate K56-2 (pCPI303-313) respectively. WT is K56-2 (pCPI301) and the vector control is K56-2 (pMS402).

Lewenza et al. identified a potential *cep *box sequence upstream of *cepI *[11]. Weingart et al. demonstrated using DNAaseI footprinting of the *cepI *promoter that CepR protected a region of DNA that corresponded to the predicted *cep *box [20]. To confirm that the *cep *box is required for *cepI *transcription, eleven mutations, each with a 4 bp substitution, were introduced into the region -59 bp to -18 bp from the transcriptional start site of *cepI *(Fig. 1A). *Bam*HI-*Xho*I fragments containing the mutations were subcloned into pMS402 directly upstream of the promoterless *luxCDABE *operon [29]. The *luxCDABE *fusions (pCPI302 to pCPI313) were introduced into K56-2 and expression determined by measuring luminescence (Fig. 1B). The K56-2 *cepI::luxCDABE *fusions with mutations within the 24 bp inverted repeat (pCPI304-310) had luminescence levels below 20% of the wild type K56-2 (pCPI301), whereas promoter fusions containing mutations flanking the inverted repeat (pCPI303, and pCPI311-314) expressed at levels either similar to or higher than wild-type.

### Identification of CepR regulated genes by transposon mutagenesis

Nine Tn*5*-OT182 transposon insertion mutants in K56-I2 were identified with differences in β-galactosidase activity on TSB-DC agar with AHL extract and TSB-DC agar without AHL extract. Expression of β-galactosidase activity was increased in the presence of OHL in six mutants and, expression was decreased in three mutants. To locate the Tn*5*-OT182 insertions in these mutants, the flanking genomic DNA was cloned, sequenced and the sequence obtained was used to search the *B. cenocepacia *J2315 genome with BlastN to identify the gene containing the insertion (Table 1). A total of 7 distinct genes in 5 regions of the genome were identified. K56-I2-P12, K56-I2-2PB2 and K56-I2-P9 had three distinct insertions within a few hundred base pairs of each other. The P12 transposon inserted into a hemin specific ATPase similar to the *phuV *gene of *Pseudomonas aeruginosa *involved in heme iron acquisition [30]. The *phuV *homolog was predicted to be in an operon with *phuR *and *phuSTUV *homologs to of *P. aeruginosa*. The *phuR *gene has been shown to be positively regulated by quorum sensing in *P. aeruginosa *[31]. The insertion in K56-I2-2PB2 transposon was also located in *phuV*; however, in this case the *lacZ *fusion was in the opposite orientation to that of the gene. K56-I2-P9 had an insertion in a hypothetical protein which appears to be in an operon and located directly downstream of a *pbp1 *homolog. K56-2-P1 and P2 were sibling insertions within a predicted acyltransferase that may be involved in lipid metabolism (COG1835). Directly upstream of the acyltransferase is a class D β-lactamase, likely an oxacillin hydrolase. The insertion in K56-I2-P3 was located in a gene, subsequently designated *scpB*, which belongs to the serine-carboxyl proteinase family [32]. K56-I2-P5 and K56-I2-P10 contained insertions located in *aidA*, which was also identified in the transposon mutagenesis screen used by Weingart et al. [20]. K56-I2-NB12 contained an insertion in an ORF that has a conserved domain (COG4774) shared with several outer membrane receptors involved in uptake of catechol siderophores, although the other genes flanking this insertion do not appear to be involved in iron acquisition. The insertion in K56-2-2PB2 did not appear to be in a gene. This insertion may result in creation of an artificial promoter-*lacZ *fusion or influence expression of a regulatory RNA.

**Figure 2:**
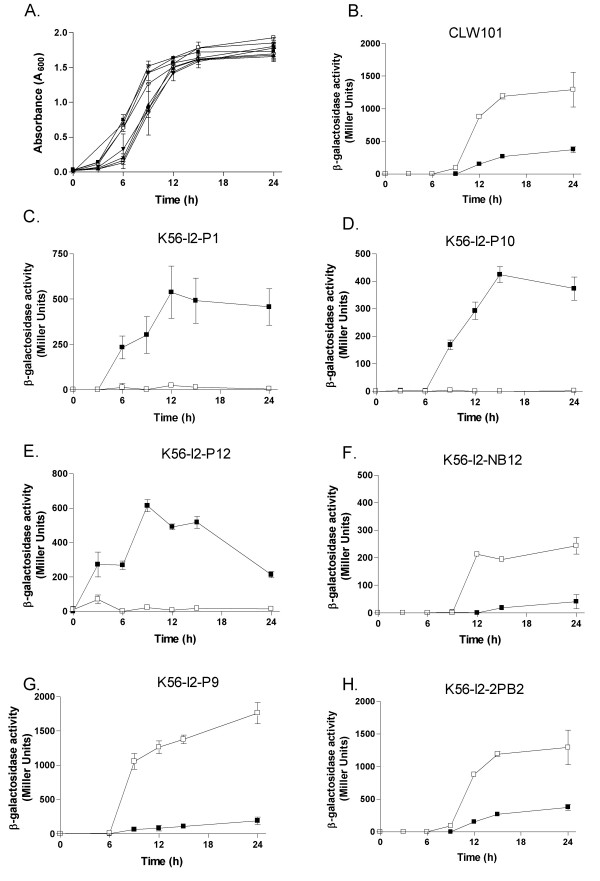
**Effect of OHL on β-galactosidase activity in K56-I2 Tn*5*-OT182 mutants**. A: Growth curves for strains shown in panels B-H. (●) CLW101; (▲) K56-I2-P1; (▼) K56-I2-P3; (□) K56-I2-P9; (◊) K56-I2-P10; (■) K56-I2-P12; (○) K56-I2-NB12; and (*) K56-I2-2PB2. Panels B-H: β-galactosidase activity with (■) and without (□) OHL. Fifty μl of OHL obtained from extracts from a 50 ml culture purified by FPLC and resuspended in 1 ml were added to 10 ml broth. This volume of OHL was shown to restore *cepI *expression to maximum levels. B: CLW101, C: K56-I2-P1, D: K56-I2-P10, E: K56-I2-P12, F: K56-I2-NB12, G: K56-I2-P9 and H: K56-I2-2PB2.

To confirm the observations in the plate assay, expression of the unique AHL responsive *lacZ *fusions was examined over a 24 hr time course in the presence and absence of OHL extract. The growth rates for each mutant were similar to the parent strain K56-I2 (Fig. 2A), indicating that the insertions did not result in growth defects that might influence *lacZ *expression. Expression of the Tn5-OT182 fusions in K56-I2-P1 (Fig. 2C) and K56-I2-P10 (Fig. 2D) were similar to that observed for a *cepI-lacZ *fusion (Fig. 2B). There was little expression in the absence of OHL and expression increased in the presence of OHL. The expression of the K56-I2-P12 fusion was also increased by the presence of OHL in the culture medium but expression started slightly earlier in growth and decreased after 10 hr (Fig. 2E). Three of the insertions appear to be negatively regulated by *cepR *since their expression was higher in the absence of OHL and decreased markedly when OHL was added to the culture medium (Fig. 2FGH). Positive regulation of β-galactosidase activity was observed for the K56-I2-P3 insertion in the presence of AHL on the plate assay; however, this fusion expressed very poorly in liquid medium (data not shown). When K56-I2-P3 grown on agar plates was analyzed for β-galactosidase activity, expression was significantly higher in cultures from plates supplemented with AHL (data not shown).

**Figure 3:**
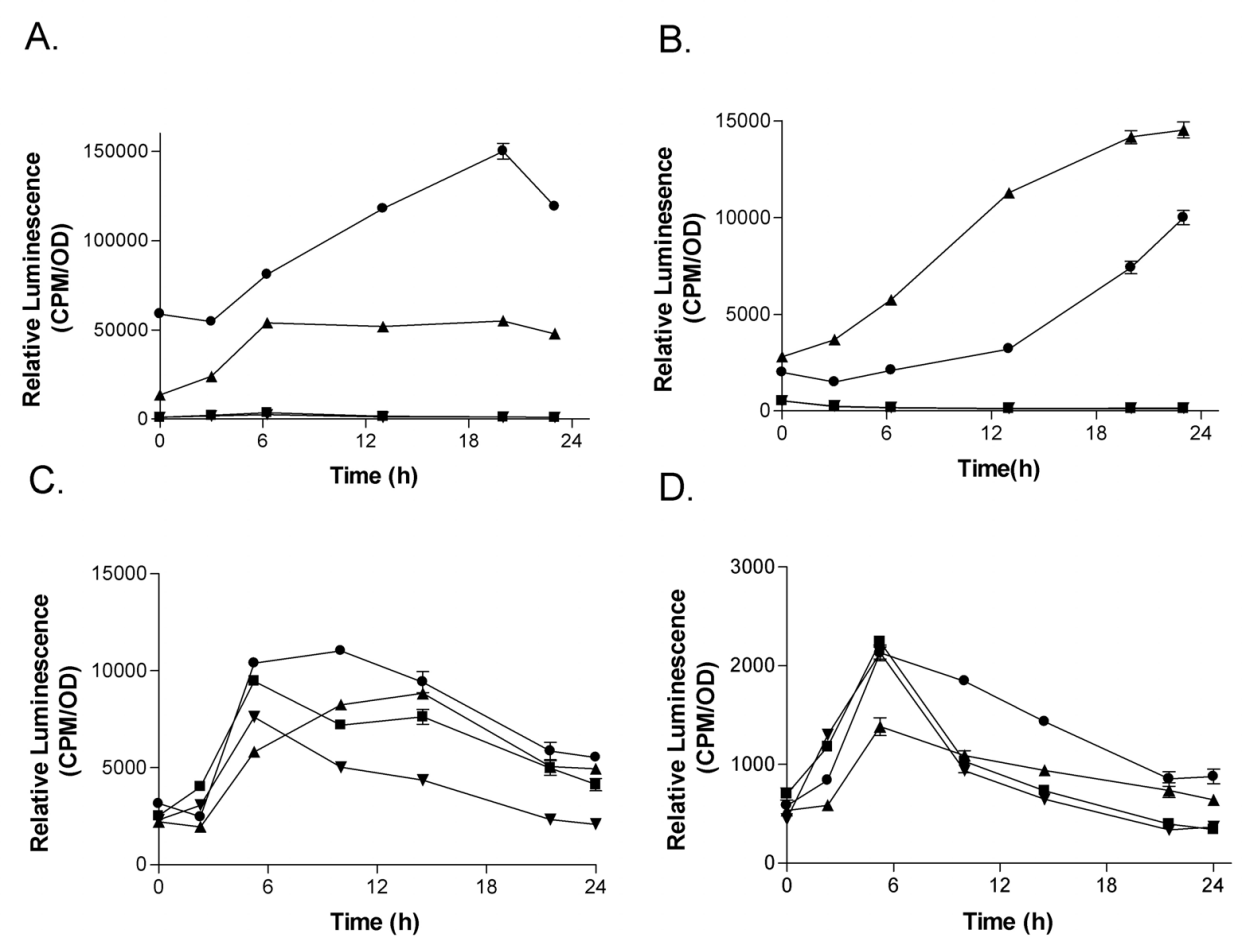
**Expression of promoter::*luxCDABE *fusions for OHL responsive genes identified by K56-I2 Tn*5*-OT182 mutagenesis**. The promoter fusions in pMS402 were introduced into strains K56-2 (●), K56-dI2 with no OHL (▼), K56-dI2 with 25 nM OHL (▲) and K56-R2 (■). Strains were grown in triplicate in 96 well microtitre plates for 24 hours. Luminescence and optical density were measured at various timepoints and the activity of the promoter was calculated as CPM/O.D. A. pCPI301 (*cepI*), B. pAID301 (*aidA*), C. pAYL301(acyltransferase), and D. pHMV301(*phuR*).

The predicted promoter regions for the three positively regulated genes containing the Tn*5*-0T182 insertions, *phuV*, *aidA *and the acyltransferase, were cloned into pMS402 and expression of the resulting promoter-*luxCDABE *fusions was determined in K56-2, K56-R2 (*cepR*) and K56-dI2 (*cepI*) with and without OHL in the medium. The *aidA *promoter fusion, pAID301, had an expression pattern similar to the *cepI *promoter with significant activity in K56-dI2 only when OHL was added to the medium (Fig. 3A and 3B). This expression pattern was similar to the chromosomal Tn*5*-OT182 *lacZ *fusion. Expression of the acyltransferase was increased in K56-dI2 in the presence of OHL; however, expression of this fusion in K56-R2 was intermediate between that in K56-dI2 and the parent strain (Fig. 3C). The *phuV *homolog was predicted to be in an operon with the promoter upstream of a *phuR *homolog and therefore the *phuR *promoter was cloned into pMS402. Expression of the *phuR *promoter was similar to K56-2 until early stationary phase where expression was significantly lower in K56-R2 and K56-dI2 in the absence of OHL (Fig. 3D). Expression of *phuR::luxCDABE *was slightly enhanced in the presence of OHL in stationary phase. The pattern of expression of the *phuR::luxCDABE *was similar to that of the *phuV::lacZ *chromosomal fusion (compare Fig. 2E and Fig. 3D). Expression of the *scpB *promoter was very weak in both the presence and absence of OHL suggesting different growth conditions are required for *scpB *expression (data not shown).

### Construction of the first generation *cep *box motif and search of the *B. cenocepacia *genome for match sequences

To identify a consensus *cep *box motif to search the *B. cenocepacia *genome for potential CepR regulated genes, promoter regions from *cepI*, *aidA*, *phuR*, the acyltransferase gene identified in K56-I2P2, *scpB*, and *zmpA*, which was previously shown to be CepR regulated [16], were analyzed using MEME to identify common motifs. Only positively regulated promoters were analyzed in case there were differences in *cep *box consensus sequences for positively and negatively regulated promoters. A motif that recognized the defined *cep *box upstream of the *cepI *gene was identified using these promoters as the input file (Table 2). The motif included bp 2–19 of the 24 bp palindrome required for transcription that contained the *cep *box for the *cepI *promoter. A single copy of the motif was found in all six of the promoters submitted. The most conserved nucleotides in the 18 bp motif were position 2 (T), 6 (A), 9 (G) and 18 (T). The position specific scoring matrix was then used to search the *B. cenocepacia *J2315 genome using the MAST program. The search returned 148 hits (numbered consecutively starting from MST001) including the 6 original input sequences (data not shown). The surrounding sequence for each hit was annotated and 49 were located upstream of predicted ORFs. The remaining hits were either within the coding sequence of an ORF or found in non-coding regions.

**Figure 4:**
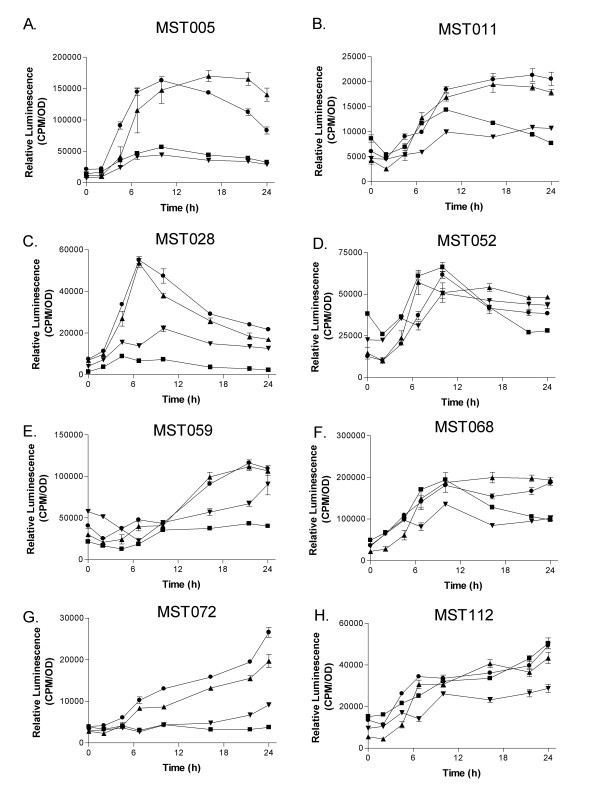
**Expression of promoter::*luxCDABE *fusionsidentified in the first *cep *box motif screen**. The promoter fusions in pMS402 were introduced into strains K56-2 (○), K56-dI2 with no OHL (▼), K56-dI2 with 25 nM OHL (▲) and K56-R2 (■). A. MST005, B. MST011, C. MST028, D. MST052, E. MST059, F. MST068, G. MST072, H. MST112. Strains were grown in triplicate in 96 well microtitre plates for 24 hours. Luminescence and optical density were measured at various timepoints and the activity of the promoter was calculated as CPM/O.D.

To determine if the putative *cep *box sequences identified were potentially involved in CepR regulation of downstream genes, eight of the promoter regions identified that were located within 40–250 bp upstream of a predicted ORF were cloned into pMS402 and expression of the resulting *luxCDABE *fusions was compared in K56-2, K56-dI2 and K56-R2. The three matching motifs with the lowest E-values and five arbitrarily selected motif matches were selected for analysis. When the motifs were located between two putative divergent promoters, one promoter region was chosen for further analysis. The predicted promoters containing putative *cep *box motifs were located upstream of the following orfs: BCAL0340, a TPR repeat protein (MST005); BCAL0715, a LysR-type transcriptional regulator (MST011); BCAL1354, a conserved hypothetical protein (MST028); BCAL2739, *fusA *(MST052); BCAL3191, *caiA *(MST059); BCAM0009, a transcriptional regulator (MST068); BCAM077, hydroxylase (MST072); and BCAM1943, a transcriptional regulator (MST112). The *luxCDABE *fusions containing the MST005, MST011, MST028, MST059 and MST072 sequences had expression patterns similar to *cepI *in that expression was higher in K56-2 than in K56-dI2 or K56-R2 and expression was increased in K56-dI2 in the presence of OHL (Fig. 4A,4B,4C,4E and 4G), although expression varied for some fusions depending on the stage of growth. For example, expression of the MST028 fusion peaked at 6 hours and decreased over the remainder of the assay (Fig. 4C). Expression of MST068 was only decreased in K56-R2 in stationary phase although expression was lower in K56-dI2 than in K56-2 and expression in K56-dI2 increased when the medium was supplemented with OHL (Fig. 4F). MST112, did not appear to be affected by the *cepR *mutation although expression was lower in K56-dI2 without OHL (Fig. 4H). MST052 did not demonstrate any regulation by CepR in the conditions examined (Fig. 4D).

### Construction of the second generation *cep *box motif and search of the *B. cenocepacia *genome for potential *cep *boxes

**Figure 5:**
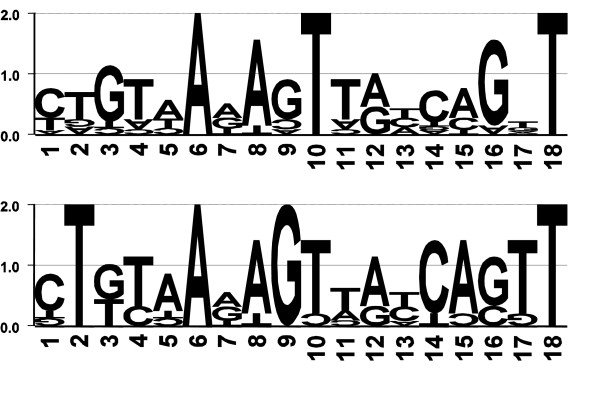
**Graphical representation of the *cep *box consensus sequence**. Nucleotide sequence logos are derived from the sequences in Table 2. The relative sizes of the letters indicate their likelihood of occurring at a particular position. The upper logo is based on the six sequences used in the first generation consensus search and the lower logo is based on the ten sequences used in the second generation consensus search.

To improve the specificity of the *cep *box motif the six promoters with cep box motifs identified by the MAST program with expression patterns similar to that expected for *cepIR *regulated genes (MST005, MST011, MST028, MST059, MST068 and MST072) were used with the promoters for *cepI*, *phuR*, *aidA *and *zmpA *to generate a second generation *cep *box consensus motif using MEME (Table 2). The promoters for *scpB*, the acyltransferase, MST052 and MST112 did not share the same expression pattern, and therefore were not included. The resulting second generation *cep *box had the same sequence as the original motif; however the specific score for each position had changed (Fig. 5). The most conserved residues in the second generation motif were in positions 6 (A), 8 (A), 10 (T), 16 (G) and 18 (T).

**Figure 6:**
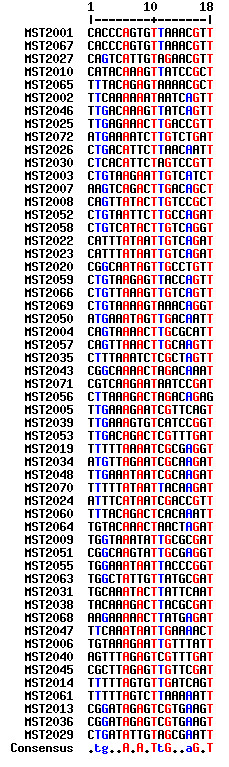
**Alignment of the putative *cep *box sequences**. The MST sequences listed are described in Table 3. Bases conserved in at least 70% of the sequences are shown in red and indicated by an upper case letter in the consensus sequence at the bottom of the alignment, and those conserved in at least 50% of the sequences are shown in blue and indicted by a lower case letter in the consensus sequence. Other bases are indicated in black.

The new PSSM file was used to search the *B. cenocepacia *J2315 genome, resulting in 72 sequences matching the motif. Fifty-five of these matches (76%) were potentially within a promoter region although it must be noted that the transcriptional start sites of these genes have not been experimentally determined. The genes or operons predicted to be downstream of these matching sequences are listed in Table 3. Both MST designations are included in Table 3 for the six first generation MSTs used in the second generation motif search. Several of the *cep *boxes identified in the second search had more significant E-values than at least one of the input sequences (data not shown). A *cep *box was identified upstream of *cepR *(MST2058), using the second motif. This was the only gene previously determined to be regulated by CepR identified. MST112, which was identified with the first motif, but did not appear to be CepR regulated (Fig. 4H), was not identified with the second motif. Potential *cep *box sequences were identified on all three chromosomes and the plasmid, suggesting that CepR regulated genes are distributed throughout the genome. Genes downstream of promoters containing *cep *boxes were classified into seven categories: cell surface or membrane protein genes, genes encoding hypothetical proteins, phage genes, regulatory genes, genes involved in secretion or transport, and genes encoding proteins of unknown function (Table 3). In ten cases the putative *cep *boxes were located between predicted divergent promoters. In these situations orfs located both downstream and upstream of the *cep *box are included in Table 3 since it would be possible that *cepR *regulates genes in both directions. An alignment of the putative *cep *boxes for each of the MST sequences listed in Table 3 is shown in Fig. 6. The most conserved residues are in position six (A), eight (A), ten (T), sixteen (G) and eighteen (T) which correlates with the motif used in the MEME input file. Further studies are needed to determine if the genes downstream of these predicted promoters and *cep *box motifs are regulated by CepR.

## Discussion

In this study we used a computational genome screen and experimental approaches to identify *cepR *regulated genes in *B. cenocepacia*. Transposon mutagenesis was used to identify OHL responsive genes in an approach similar to that described by Weingart et al[20]. Since we had previously determined that genes involved in production of the siderophore ornibactin were *cepIR *regulated [14], we performed our screen in low iron medium in an attempt to identify other iron regulated genes that were responsive to OHL. We also had previously determined that *cepR *could both positively and negatively regulate gene expression, and therefore, the transposon library was screened for insertion mutants in which β-galactosidase activity was either turned on or off in the presence of exogenous AHLs. Four unique positively regulated and three negatively regulated *lacZ *fusions were identified. We identified two genes potentially involved in iron transport, a putative outer membrane receptor (BCAM1187) and *phuV*, a hemin ATP binding protein (BCAM2630). Interestingly, expression of the outer membrane receptor gene was negatively influenced by OHL, whereas *phuV *expression was positively influenced.

In a screen of approximately 25,000 transposon mutants we only identified six loci with AHL responsive genes. The screening assay was dependent on the visual identification of colonies that were either blue or white in the presence or absence of AHL on medium with X-gal. Although we were able to detect as little as two-fold differences in expression with this assay, we would not detect gene fusions expressed in both the presence and the absence of AHL since we did not attempt to identify mutants with varying shades of blue. For example, although CepR positively regulates *zmpA*, the CepIR system is not required for its expression since *zmpA *is expressed at low levels in the absence of AHL and in *cepI *or *cepR *mutants [16]. The *lacZ *fusions in the positively regulated genes identified with transposon insertions were only expressed at significant levels in the presence of OHL. The three negatively regulated fusions had very low expression in the presence of OHL (Fig. 2). It was surprising that we did not identify *cepI *since CepR tightly regulates *cepI *expression [14] and *cepI *appeared to be a hotspot for transposon insertions in the study by Weingart et al. [20]. The *aidA *gene which is tightly regulated by *cepIR *was identified in both transposon screens, as well as the proteomics and promoter trap approaches [17,19].

Lewenza et al [11] identified a putative CepR binding site in the *cepI *promoter. During the course of this current study it was reported that CepR directly interacted with a *cep *box that overlapped this region and directly bound to a *cep *box within the *aidA *promoter [20]. We demonstrated using site directed mutagenesis of the *cep *box region that a 24 bp sequence that contained the *cep *box was required for *cepI *expression. All *cepI*::*luxCDABE *promoter fusions with mutations in the 24 bp *cep *box had levels of expression less than or equal to 20% of K56-2 (pCPI301). Similar mutations constructed flanking the *cep *box had either no effect or in one case increased transcription.

The use of bioinformatics to identify CepIR regulated genes has several advantages that are complementary to the experimental methods used to search for CepIR regulated genes. Procedures such as transposon mutagenesis, promoter libraries, microarray analysis or proteomics are dependent on the transcription and expression levels of the genes and on the conditions used in the study. Furthermore, the genes and proteins identified by these approaches may be regulated directly or indirectly by CepR. The use of a motif in a genome-wide search for CepIR regulated genes may identify niche specific genes that may only be expressed in certain conditions. Identification of a *cep *box motif may also be used to predict whether CepIR genes are directly regulated by interaction with CepR at the promoter or indirectly by CepR interaction with a promoter for an intermediate regulatory gene. In fact, 14 of the 55 putative *cep *boxes identified were in the predicted promoter regions for regulatory genes. We are currently characterizing some of these regulatory genes to confirm that they are *cepR *regulated and to determine their regulatory properties.

When searching the genome using the first generation *cep *box motif we identified some sequences that were not identified with the refined motif used in the second screen of the genome (data not shown). It is possible that these genes are regulated by CepR but have less conserved *cep *box sequences. Of the eight promoter-*lux *fusions constructed from sequences identified in the first generation search, six were determined to have *cepR *regulated expression. There was no difference between the expression of the pMST112 in K56-R2 and K56-2; however, luminescence was increased in K56-dI2 in medium with OHL. The MST112 motif was not detected in the second *cep *box motif, suggesting that this BCAM1943 may not be *cepR *regulated. Mutations in *cepI *or *cepR *did not influence the expression of pMST052 (BCAL2739). Although this promoter region was excluded from the group used to generate the second motif, this potential *cep *box was also detected in the second search (MST2035). It is possible that BCAL2739 is, in fact, CepR regulated in different medium or growth conditions.

Interestingly, the MEME program identified a *cep *box motif farther upstream of the *aidA cepR *binding site identified by Weingart et al. [20]. It is possible that there is more than one CepR binding site upstream of *aidA*. The additional site might contribute to its tight regulation by CepR and dependency on OHL for expression, features that may have resulted in *aidA *being detected in all of the approaches to date to identify CepR regulated genes.

We identified a *cep *box in the *cepR *promoter region that contains all of the most conserved bases. We have previously shown that *cepR *negatively regulates itself [14]. This is the first confirmed negatively regulated gene identified in the motif search.

It is difficult to compare the extent of overlap between the genes identified using the bioinformatics approach to those identified by Aguilar et al. [17] and Weingart et al. [20] since the same annotation of the J2315 sequence was not used, although Aguilar et al. identified in addition to *aidA*, a *lysR *regulator and a putative short chain dehydrogenase which may be the same ones we identified. Concurrent with this study, we employed a random promoter library approach to identify promoter::*lux *fusion clones that were differentially expressed in the presence or absence of OHL in K56-dI2 [33]. Of the 86 promoter clones identified, surprisingly only 4 genes overlapped between the two approaches, BCAM0009, BCAM0010, *cepI *and *zmpA*. A putative *cep *box was identified in the promoter regions of 30/89 OHL responsive genes from the promoter library, but generally with only 50–60% identity to the *cep *box consensus identified in this study. Therefore, these would not have been identified with the stringency employed in the search. It is surprising that more genes that were identified using the *cep *box motif were not found in the promoter library, although the promoter library also lacks other known CepR regulated genes indicating that it is not complete. Some of the genes with *cep *boxes may not be expressed in the conditions used to screen the library.

Strains of *B. cenocepacia*, including K56-2, that contain the cenocepacia island (cci) have a second set of quorum sensing genes [34]. CciI is an AHL synthase that produces predominantly HHL and small amounts of OHL. CciR is the transcriptional regulator. CciIR are co-transcribed and *cepR *is required for *cciIR *expression [35]. Little is currently known about the regulatory targets of *cciIR*, although the zinc metalloproteases *zmpA *and *zmpB *are regulated by *cciIR*, and CciR negatively regulates *cepI *[35,36]. There is no apparent *cep *box upstream of *cciIR*; however, there is one located within the coding sequence 13 bp downstream of the predicted start codon. This putative *cep *box TTGCTGAAGTTGTTCGGT lacks the conserved A in position 6 present in all the sequences in Table 3 but contains the other conserved bases. It is currently unknown whether CciR binds to a similar site as CepR, but we have determined that some *cepR *regulated genes are not regulated by *cciIR *(data not shown). It is possible that some of the *cep *boxes we have identified might be CciR binding sites. Further studies are in progress to explore the regulatory relationships between these two quorum sensing systems in *B. cenocepacia*.

## Conclusion

We have identified several new CepR regulated genes using transposon mutagenesis and *lux *promoter fusions. We have also used a *cep *box consensus sequence to identify several genes or operons potentially regulated by CepR. To confirm that these genes are regulated by *cepR *or possibly *cciR*, experimental approaches such as transcriptional fusions, microarrays, or demonstration of direct binding of CepR to their promoter regions will be required. These studies reveal a significant number of genes that may be further studied to increase our understanding of the CepR regulon.

## Methods

### Reagents, bacterial strains and culture conditions

Unless otherwise stated all molecular biology reagents were purchased from Invitrogen Life Technologies (Burlington, Ontario) and all chemicals purchased from Sigma Chemical Co. (St. Louis, Mo.). The strains and plasmids used in this study are listed in Table 4. For genetic manipulations, *B. cenocepacia *and *Escherichia coli *strains were grown at 37°C in Luria-Bertani (LB) broth (Invitrogen) or on 1.5% LB agar plates. Concentration of antibiotics in selective medium for *E. coli *were 100 μg/ml ampicillin, 1.5 mg/ml trimethoprim and 15 μg/ml tetracycline, and for *B. cenocepacia *were 200 μg/ml tetracycline and 100 μg/ml trimethoprim. For transcription assays, *B. cenocepacia *strains were grown in tryptic soy broth (TSB, Difco, Detroit, Mich.) or TSBD-C [37].

### AHL extraction and OHL purification

AHLs were extracted from culture supernatants of K56-2 as previously described [14]. The extract from 50 ml culture fluid was resuspended in 1 ml distilled water and 20 μl aliquots of this stock solution were spread onto agar plates to screen for mutants in which *lacZ *expression was altered in the presence of AHL. This quantity of AHL extract was found to restore wild-type protease activity to *B. cenocepacia *K56-I2 as indicated by the zones of clearing observed on skim milk plates. OHL was purified from culture supernatants of *B. cenocepacia *K56-I2 (pSLS225), a strain that carries the *cepI *gene *in trans *as previously described [38].

### Molecular biology and sequence analysis

DNA manipulations were performed generally as described by Sambrook *et al*. [39]. T4 DNA ligase was purchased from Promega Corporation (Madison, WI) and New England Biolabs Inc. (Beverly, MA). Custom oligonucleotides were synthesized by Invitrogen Life Technologies. DNA sequencing was performed at the Univeristy of Calgary Core DNA Services (Calgary, Canada) using an ABI1371A DNA sequencer or at Macrogen Inc. (Seoul, Korea) on an ABI3730 XL automatic DNA sequencer.

### Transposon mutagenesis

Mutagenesis of *B. cenocepacia *K56-I2 (Tp^r^) with Tn*5*-OT182 was performed as described by Lewenza *et al*. [11]. Tn*5*-OT182 is a self-cloning transposon with a promoterless *lacZ *gene that is transcribed from the promoter of a host gene when it is fused in the direction of transcription [40]. Transposon insertion mutants were picked using a robot (Norgren Systems, Palo Alto, CA) into Becton Dickenson microtest flat bottom polystyrene 96 well microtiter plates containing 200 μl medium per well and grown overnight at 37°C with shaking at 200 rpm. Cultures were stamped onto TSBD-C (200 μg/ml tetracycline, 100 μg/ml trimethoprim and 40 μg/ml X-gal) agar with and without the addition of AHL extract and grown for 48 hours at 37°C. β-galactosidase expression was visually monitored at 24 and 48 hours for differences in blue color. Approximately 25,000 tetracycline and trimethoprim resistant transposon insertion mutants from five independent mutagenesis experiments were screened. Positively regulated insertion mutants appeared blue in the presence of AHL and X-gal and white in the absence of AHL. The reverse is true in the case of negatively regulated genes. Nine mutants exhibiting reproducible differences in AHL dependent β-galactosidase expression were chosen for further characterization. The DNA flanking the Tn*5*-OT182 insertions was self-cloned from *Xho*I or *Eco*RI digests of genomic DNA and sequenced using oligonucleotides OT182-LT and OT182-RT [41].

### Construction of *cepI *promoter mutations

The Altered Sites^® ^II *in vitro *Mutagenesis System (Promega) and the Quick Change^® ^Site-Directed Mutagenesis Kit (Stratagene, La Jolla, CA) were used to create mutations spanning the proposed *cep *box in the *cepI *promoter (Fig. 2). The template used with the Altered Sites^® ^II *in vitro *Mutagenesis System was created by ligating a *Bam*HI-*Xba*I fragment containing the *cepI *promoter region from pCPI101 into pALTER-*Ex*1 (pCPI201). The Altered Sites^® ^II System was used with mutagenic oligonucleotides CepBx103-106 (Table 5). These oligonucleotides were 5'-phosphorylated using T4 DNA Kinase (Promega) and annealed to single stranded DNA prepared according to the manufacturers instructions from cultures of JM109 F' (pCPI201). The remaining mutagenic oligonucleotides were used with plasmid pCPI101 and the Stratagene Quick Change^® ^Site-Directed Mutagenesis Kit. Mutagenic oligonucleotides were designed with 4 base pair substitutions that resulted in the introduction of a new restriction enzyme site (Table 5). Mutations were confirmed by restriction enzyme analysis and sequencing. To construct the *cepI::luxDCABE *fusions, the mutated promoter regions were excised from pCPI101 and pCPI201 by digestion with *Bam*HI-*Xho*I and ligated into the *Bam*HI-*Xho*I site of pMS402.

### *In vitro *transcription assays

Putative promoters identified in this study were PCR amplified using the primers listed in Table 5 from K56-2 genomic DNA and cloned into the vector PCR2.1^®^-TOPO. The promoters were excised from the PCR2.1^®^-TOPO clones using *Bam*HI-*Xho*I and ligated into pMS402 to create plasmids pCPI301, pPHU301, pAYL301, pSCP301 and pAID301, respectively. The eight promoters identified in the first genome search for *cep *box motifs were cloned using the primers listed in Table 5 for each MST promoter as described above and named pMST005, pMST011, pMST028, pMST052, pMST059, pMST068, pMST072, and pMST112, respectively.

Five ml overnight cultures of K56-2, K56-dI2 and K56-R2 hosting the *luxDCABE *fusions were grown in TSB supplemented with 100 μg/ml trimethoprim to maintain pMS402. Overnight cultures were diluted with TSB to an A_600 _of 0.05 and aliquots of 150 μl were placed in wells of 96 well clear bottom plates (Costar, Corning Incorporated, Corning, NY). The plates were covered and incubated at 37°C with shaking and the luminescence and absorbance was measured in a Victor^2^™ multilabel counter at various intervals for 24 hours. Each strain was assayed at least three times in triplicate.

### Bioinformatics

Nucleotide sequence obtained from DNA flanking the transposon insertions was used with BLASTN to determine the location of the insertion in the unpublished genome sequence of *B. cenocepacia *J2315 [42], a strain of the same lineage as K56-2. Homologues of open reading frames were predicted using BLASTP [43]. Potential promoter elements were identified using BPROM [44]. The *cep *box consensus sequence was predicted by analyzing the promoter regions of selected positively regulated genes with the motif discovery tool MEME [45]. The MEME program [46] represents motifs as position-dependent letter-probability matrices which describe the probability of each possible letter at each position in the pattern. The output from the MEME program provides a position-specific scoring matrix (PSSM) for the predicted motif. The PSSM for the predicted *cep *box consensus sequence was used to search the *B. cenocepacia *J2315 genome with the motif alignment search tool MAST [45,47]. The *cep *box motifs identified by MAST were also aligned using Multalin [48,49].

## Authors' contributions

CC designed the *cep *box mutagenesis, performed the analysis of the *cep *box consensus sequence and screening of the genome, contributed to the promoter fusion expression experiments, and helped draft the manuscript. EL performed the transposon mutagenesis, expression experiments on the mutants, *cep *box alignments and helped draft the manuscript. MV constructed *cep *box mutants, analyzed genome sequence data, contributed to promoter fusion assays, and helped draft the manuscript, PL cloned MST promoters and performed *lux *fusion assays, PS participated in the experimental design and data analysis, coordinated the study and drafted the manuscript. All authors read and approved the final manuscript.

## Figures and Tables

**Table 1:** OHL responsive genes identified by Tn*5*-OT182 mutagenesis of K56-I2

Transposon Mutant	Orf^a^	Predicted start codon^b^	Location of insert^b^	Gene/domain homology^c^	OHL effect on expression^d^
K56-I2-P1, K56-I2-P2	BCAM0392	2:445357	2:444971	COG1835: Predicted acyltransferases	+
K56-I2-P3	BCAM0957	2:1062298	2:1060868	*scpB*: serine-carboxyl proteinase precursor	+
K56-I2-P5, K56-I2-P10	BCAS0293	3:328037	3:328810	*aidA*	+
K56-I2-P9	BCAM2631	2:2981279	2:2980753	COG2860: predicted membrane protein	-
K56-I2-P12	BCAM2630	2:2979794	2:2980345	*phuV*: hemin specific ATP-binding protein	+
K56-I2-2PB2	no gene		2:2980336		-
K56-I2-NB12	BCAM1187	2:1298085	2:1297891	COG4774, Outer membrane receptor	-

^a ^Open reading frame designation from the unpublished annotation of the *B. cenocepacia *J2315 genome.

^b ^Locations reported as chromosome:nucleotide.

^c ^Gene and domain homologies determined using BLASTP.

^d ^Effect of OHL on expression of the *lacZ *fusion created by insertion of the transposon. +, positive regulation or greater expression in the presence of OHL; -, negative regulation or lower expression in the presence of OHL.

**Table 2:** Identification of a *cep *box consensus motif.

Gene	*p*-value^b^	direction	Sequence^c^	bp to ORF^d^
First Generation Motif Sequences^a^				

*cepI*	2.23E-11	+	CACC**CTGTAAGAGTTACCAGTT**ACAGGCTC	72
*phuR*	6.53E-10	+	TACA**CTGTTAAAGTTGTCAGTT**GCCTTTCA	116
*aidA*	2.24E-09	-	GAAG**CTGTAAAAGTAAACAGGT**CGGGAAAA	159
*zmpA*	2.60E-09	+	TCTT**GTTTAAAAGTCATCACTT**GATGCATT	54
Acyltransferase	1.13E-07	-	AGGG**CTTCAAGTGTAACTCCTT**GGAAAGGT	3
*scpB*	1.25E-07	-	CCAG**TTTCCATAGCTGTCAGTT**CTGACAAC	115
**consensus**			**CTGTAAAAGTTACCAGTT**^e^	

Second Generation Motif Sequences^f^				

*cepI*	4.79E-11	+	CACC**CTGTAAGAGTTACCAGTT**ACAGGCTC	72
*phuR*	6.11E-11	+	TACA**CTGTTAAAGTTGTCAGTT**GCCTTTCA	116
MST072	4.01E-09	+	AAAA**TTGACAAAGTTATCAGTT**ATGACTTT	56
*aidA*	2.16E-08	-	GAAG**CTGTAAAAGTAAACAGGT**CGGGAAAA	159
MST028	5.32E-07	-	CTTT**CGGCAATAGTTGCCTGTT**TCGATTGA	140
*zmpA*	2.60E-09	+	TCTT**GTTTAAAAGTCATCACTT**GATGCATT	54
MST005	1.24E-06	+	CAAC**CAGTAAAACTTGCGCATT**CCGGTCGA	206
MST068	1.60E-06	-	CGTT**CGCTTAGAGTTGTTCGAT**ATTTCGAA	138
MST011	1.80E-06	+	TGTC**AAGTCAGACTTGACAGCT**TGTAAAGG	76
MST059	2.41e-06	+	ATGG**TTGAAAGTGTCATCCGGT**GCTACACT	118
**consensus**			**CTGTAAAAGTTACCAGTT**^g^	

^a ^The promoter regions of 6 genes experimentally determined to be positively regulated by CepR used to search for common motifs with the MEME (Multiple EM for Motif Elicitation) program.

^b ^The *p*-value of a site is computed from the the match score of the site with the position specific scoring matrix for the motif. The *p*-value gives the probability of a random string (generated from the background letter frequencies) having the same match score or higher. (This is referred to as the **position *p*-value **by the MAST algorithm.)

^c ^The boxed region represents the region determined to be required for *cepI *expression as determined in Fig. 1. Bold lettering represents the motif predicted by the MEME program. In the case of *cepI *the motif matches the CepR binding site [20].

^d ^The number of base pairs to the start codons predicted by alignment with homologous genes.

^e ^Underlined bases are conserved in at least 4 of 6 sequences.

^f ^The promoter regions of 10 genes experimentally determined to be positively regulated by CepR used to search for common motifs with MEME.

^g ^Underlined bases are conserved in at least 7 of 10 sequences.

**Table 3:** *B. cenocepacia *J2315 genes identified using the second generation *cep *box motif

Motif name^a^	Position^b^	bp^c^	gene^d^	Gene/domain and predicted function^e^ (Adjacent downstream genes possibly in operon)
**Cell Surface or Membrane**				

MST2008 (-)	1:806161	45	BCAL0738(-)	COG0793: Periplasmic protease; cell envelope biogenesis
MST2009 (+)	1:901874	295	BCAL0831 (+)	*phaP*: phasin-like protein
MST2031 (-)	1:2662911	104	BCAL2406(-)	COG0859, *rfaF*, LPS heptosyltransferase (*rfa*L,*rfaG*; LPS biosynthesis genes)
MST2048 (-)	2:211218	106	BCAM0183 (+)	COG3468, autotransporter type V secretion, *shdA *homolog: adhesin
MST2050 (+)	2:1129604	172	BCAM1015(-)	COG3203: Outer membrane protein
MST2068 (-)	3:174253	153	BCAS0156(+)	COG1680: ampC, β-lactamase class C

**Hypothetical Protein**				

MST2014 (-)	1:1228119	131	BCAL1124 (+)	Conserved hypothetical protein
**MST2020 (+) MST028^f^**	1:1484174	140	BCAL1354(-)	COG4104: conserved hypothetical protein (*vgrG*: vgr related protein)
MST2030 (-)	1:2567308	41	BCAL2313 (+)	hypothetical protein
MST2052 (+)	2:1249946	118	BCAM1149 (+)	hypothetical protein
MST2056 (-)	2:1667312	57	BCAM1502 (+)	hypothetical protein (Chemoreceptor *mcpA*)
MST2063 (-)	2:2720454	-19	BCAM2417 (+)	hypothetical protein
MST2067 (+)	2:3070180	254	BCAM2713(-)	hypothetical protein
MST2071 (+)	3:836110	63	BCAS0753(+)	hypothetical protein

**Metabolism**				

MST2002 (-)	1:273243	21	BCAL0232 (+)	Elongation factor Tu
MST2005 (+)	1:390962	47	BCAL0358 (-)	COG0308: Aminopeptidase N
**MST2007 (+) MST011**	1:778996	101	BCAL0716 (+)	COG1250: *fadB*, 3-hydroxyacyl-CoA dehydrogenase; lipid metabolism
MST2010 (-)	1:963495	59	BCAL0886 (+)	COG0183: *paaJ*, Probable beta-ketoadipyl CoA thiolase (*caiD*; lipid metabolism)
MST2022 (-)	1:1602043	50	BCAL1448(-)	COG0525: *valS*, Valyl-tRNA synthetase
MST2023 (-)	1:1626201	104	BCAL1468(-)	COG0644: *fixC*, electron transfer flavoprotein-ubiquinone oxidoreductase
MST2027 (+)	1:2465614	32	BCAL2229(-)	Hypothetical signal peptide protein (COG3000: Sterol desaturase, lipid metabolism)
MST2029 (-)	1:2554533	153	BCAL2302(-)	COG0556,*uvrB*: Helicase subunit of the DNA excision repair complex
MST2029 (-)	1:2554533	106	BCAL2303 (+)	COG1448, *tyrB*: aspartate/tyrosine/aromatic aminotransferase
MST2034 (+)	1:2903040	55	BCAL2638 (+)	COG0165,*argH*: Argininosuccinate lyase, arginine biosynthesis
MST2035 (+) MST052	1:3009329	9	BCAL2739 (+)	COG0480, *fusA*: Translation elongation factor
MST2038 (+)	1:3351536	-15	BCAL3058 (+)	COG0043, *ubiD*: 3-polyprenyl-4-hydroxybenzoate decarboxylase (rhtB, Putative threonine efflux or homoserine/homoserine lactone efflux)
**MST2039(+) MST059^f^**	1:3488874	117	BCAL3191(+)	COG1960: *caiA*, acyl CoA dehydrogenase
MST2043 (+)	1:3745369	60	BCAL3419 (+)	COG0757: *aroQ*: 3-dehydroquinate dehydratase II
MST2045 (+)	2:11142	137	BCAM0010(+)	*kbl *homolog, AKB ligase
**MST2046 (+) MST072^f^**	2:84847	55	BCAM0077(-)	COG0654: *ubiH *or *mhpA*, hydroxylase
MST2055 (+)	2:1564008	139	BCAM1405(-)	*sacB*: Levansucrase (*sacC*: Levanase precursor)
**MST2059 (+)**^f^	2:2088113	71	BCAM1870 (+)	*cepI*: homoserine lactone synthase
MST2061(+)	2:2134837	112	BCAM1922(+)	*repA*: replication protein
MST2064 (-)	2:2839793	44	BCAM2502(-)	COG0757: *aroQ*: 3-dehydroquinate dehydratase II (*aroE*: Shikimate 5-dehydrogenase)
MST2064 (-)	2:2839793	125	BCAM2503(+)	COG3185: *hppD*, 4-hydroxyphenylpyruvate dioxygenase
MST2065 (-)	2:2938113	48	BCAM2588(-)	*menG*: putative S-adenosylmethionine:2 demethylmenaquinone methyltransferase

**Phage genes**				

MST2024 (+)	1:1735446	71	BCAL1564 (-)	Hypothetical proteins Mup46, Mup47 and Mup48 [phage tail protein]
MST2060 (+)	2:2096677	28	BCAM1879 (+)	Phage antirepressor

**Regulatory gene**				

MST2006 (-)	1:616909	88	BCAL0562(-)	COG2747, *flgM*: Negative regulator of flagellin synthesis (*flgN*; Flagellar biosynthesis/type III secretory pathway)
**MST2007 (+) MST011^f^**	1:778996	59	BCAL0715(-)	COG0583: LysR-type transcriptional regulator
MST2013 (+)	1:1085981	40	BCAL0999 (+)	COG3073: RseA; Negative regulator of sigma E activity (RseB or MucB, negative regulator for alginate biosynthesis)
MST2019 (-)	1:1437591	385	BCAL1318 (+)	COG3707, *nasR*: nitrate-and nitrite-responsive positive regulator
MST2026 (+)	1:2016418	259	BCAL1826 (+)	*gltF*: regulator of gltBDF operon, glutamate synthaseenzymes
MST2036 (-)	1:3153030	18	BCAL2871(-)	COG3073, *rseA*: Negative regulator of sigma E activity (*mucB/rseB, mucD*)
**MST2039 (+) MST059**	1:3488874	102	BCAL3190(-)	COG1414: Transcriptional regulator, IclR family
MST2040 (-)	1:3502381	36	BCAL3205(-)	COG1396: *hipB *homolog, Putative transcription regulator
**MST2045 (+) MST068^f^**	2:11142	22	BCAM0009(-)	COG1396: *hipB *homolog, Predicted transcriptional regulator
**MST2046 (+) MST072**	2:84847	58	BCAM0076(-)	COG1309: ArcR domain: Bacterial regulatory proteins, tetR family
MST2055(+)	2:1564008	64	BCAM1406(+)	COG: *aglR*, HTH-type transcriptional regulator
MST2057 (+)	2:1959876	36	BCAM1750 (+)	COG1846: Transcriptional regulator, MarR family
MST2058^f ^(+)	2:2087487	31	BCAM1868(-)	*cepR*: Transcriptional regulator, LuxR family
MST2071 (+)	3:836110	40	BCAS0752(-)	COG0583: LysR type Transcriptional regulator

**Secretion or secreted product**				

MST2003 (+)	1:351306	25	BCAL0321 (+)	COG3671: Predicted membrane protein (*tatA*, *tatB*, *tatC *secretion pathway)
**MST2004 (+) MST005^f^**	1:366026	206	BCAL0340 (+)	COG0457: TPR repeat, (*evpA *and *evpB, evpC, evpE*, *evpF*, and *evpG *virulence and possible secretion)
**MST2070(-)**^f^	3:478440	108	BCAS0409 (+)	*zmpA*: extracellular zinc metalloprotease

Transport				

MST2001 (-)	1:61816	272	BCAL0051 (+)	COG0834: ABC-type amino acid transport/signal transduction systems
**MST2066 (+)**^f^	2:2974227	115	BCAM2626 (+)	*phuR*: Haem/Haemoglobin uptake outer membrane receptor precursor (*phuS*, *phuT, phuU phuV*
MST2072 (+)	P:55610	113	PBCA053 (-)	COG1638,*dctP *homolog: TRAP-type C4-dicarboxylate transport system,

Unknown				

**MST2004 (+) MST005**	1:366026	181	BCAL0339(-)	COG3521: Uncharacterized protein conserved in bacteria
MST2025 (+)	1:1979817	274	BCAL1791 (-)	COG2606: Uncharacterized conserved protein
MST2047 (-)	2:169540	86	BCAM0148 (+)	Putative vgr-related protein (*pldA: *Phosphatidylserine/phosphatidylglycerophosphate/cardiolipin synthases)
MST2051 (-)	2:1150388	-37	BCAM1044(-)	no homology (COG1536: Flagellar motor switch protein)
MST2053 (-)	2:1467792	28	BCAM1328-329 (+)	Unknown proteins
MST2061 (+)	2:2134837	59	BCAM1921-919 (-)	no homologs
**MST2069 (+)^f^**	3:329197	160	BCAS0293(-)	*aidA*, intracellular protein of unknown function involved in nematode virulence*; *(second *aid*A)

^a ^MSTs were identified by searching the *B. cenocepacia *J2315 genome with the position specific scoring matrix (PSSM) from the second generation motif. Only genes with a motif match within a potential promoter and within 300 bp of the predicted start codon are reported.

^b ^The location center of the predicted motif is reported as chromosome:nucleotide. (+) or (-) refers to the DNA strand encoding the motif sequence. The motif names in bold were used to generate the PSSM file.

^c ^Number of base pairs between the centre of the motif and the predicted translational start site.

^d ^Open reading frame number from the unpublished annotation of the *B. cenocepacia *J2315 genome. (+) and (-) refer to the DNA strand.

^e ^Gene and domain homologies were obtained using the standard protein-protein BlastP program as described in the methods. Genes in parantheses are downstream of the first orf following the motif and may be in the same operon.

^f ^Confirmed to be CepR regulated by either *lux *or *lacZ *transcriptional fusions.

**Table 4:** Bacterial strains and plasmids used in this study.

Strain or plasmid	Description and relevant genotype	Source
*E. coli*		
DH5α	φ80d*lacZ*ΔM15 (*lacZYA-argF*) *recA1 endA gyrA96 thi -1 hsdR17 supE44 relA1 deoR U169*	Invitrogen
SM10	Mobilizing strain, RP4 *tra *genes integrated in the chromosome, Km^r^	[50]
DH10B	F^-^*mcrA *Δ(*mrr*-*hds*RMS-*mcr*BC) φ80d*lac*ZΔM15 Δ*lac*X74 *end*A1 *rec*A1 *deo*R Δ(*ara, leu*)7697 *ara*D139 *gal*U *gal*K *nup*G *rps*L λ-	Invitrogen
JM109 F'	*end*A1 *rec*A1 *gyr*A96 *thi hsd*R17 (r_k_- m_k_+) *rel*A1 *sup*E44 Δ(*lac-pro*AB) [F' *tra*D36 *pro*AB *laq*IqZΔM15]	Promega
*B. cenocepacia*		
K56-2	Cystic fibrosis respiratory isolate	[51]
K56-R2	*cepR::*Tn*5*-OT182 derivative of K56-2, Tc^r^	[11]
K56-I2	*cepI*::tp derivative of K56-2, Tp^r^	[11]
K56-dI2	*cepI *deletion mutant of K56-2	[35]
CLW101	*cepI*::*tp*::Tn*5*-OT182 derivative of K56-2, Tc^r^, Tp^r^	[20]
K56-I2-P1	BCAM03092:: Tn*5*-OT182 derivative of K56-I2, Tc^r^	This study
K56-I2-P3	BCAM0957:: Tn*5*-OT182 derivative of K56-I2, Tc^r^	This study
K56-I2-P5	BCAS0293:: Tn*5*-OT182 derivative of K56-I2, Tc^r^	This study
K56-I2-P9	BCAM2631:: Tn*5*-OT182 derivative of K56-I2, Tc^r^	This study
K56-I2-P12	BCAM2630:: Tn*5*-OT182 derivative of K56-I2, Tc^r^	This study
K56-I2-2PB2	Tn*5*-OT182 derivative of K56-I2, Tc^r^	This study
K56-I2-NB12	BCAM1187:: Tn*5*-OT182 derivative of K56-I2, Tc^r^	This study
Plasmids		
pCR^®^2.1 TOPO	PCR cloning vector, pUC ori, P*lac*, *lacZα*, Km^R ^Ap^R^	Invitrogen
pOT182	pSUP102(GM)::Tn*5*-OT182, Cm^r^, Tc^r^, Gm^r^, Ap^r^	[40]
pALTER^®^-*Ex*1	mutagenesis plasmid, Tc^r^	Promega
pSLS225	pUCP26 with 1.5 kb *Sph*I-*Kpn*I fragment containing the *cepI *gene, Tc^r^	[11]
pCPI101	pCR^®^2.1 TOPO with a 266 bp fragment containing the *cepI *promoter, Ap^r^, Km^r^	This study
pCPI201	pAlter^®^-*Ex*1 with the *Bam*HI-*Xba*I fragment from pCPI101, Tc^r^	This study
pMS402	Broad host range vector with promoterless *luxCDABE *operon, Tp^r^, Km^r^	[29]
pCPI301	pMS402 with the *Bam*HI-*Xho*I fragment containing the wild type *cepI *promoter region from pCPI101, Tp^r^, Km^r^	This study
pCPI303-313	pMS402 containing the *Bam*HI-*Xho*I fragments containing the *cepI *promoter region with the *cep *box mutations designated 303-313, Tp^r^, Km^r^	This study
pRK2013	ColE1 Tra (RK2)^+^, Km^r^	[52]
pPHU301	pMS402 containing the *phuR *promoter region	This study
pAYL301	pMS402 containing the acyltransferase promoter region	This study
pSCP301	pMS402 containing the *scpB *promoter region	This study
pAID301	pMS402 containing the *aidA *promoter region	This study
pMST005	pMS402 containing the MST005 promoter region	This study
pMST011	pMS402 containing the MST011 promoter region	This study
pMST028	pMS402 containing the MST028 promoter region	This study
pMST052	pMS402 containing the MST052 promoter region	This study
pMST059	pMS402 containing the MST059 promoter region	This study
pMST068	pMS402 containing the MST068 promoter region	This study
pMST112	pMS402 containing the MST112 promoter region	This study

**Table 5:** Oligonucelotide primers

Primer	Sequence		Restriction Site or size of product (bp)
PCR Oligonucleotides			

cepIfor	CAGGCGGCGATAGCTTG		
cepIrev	CACAGATCCGAGGACATCCA		
EXcepR3	CGGGATCCGAGAAAGAATGGAACTGCGC		*Bam*HI
EXcepR2	CGGGATCCTTGCGTCAGGGTGCTTCGATG		*Bam*HI

oligonucleotides used to clone promoters		position of 5' base^a^	Size (bp)

*aidA*	CAGATTCAATGTCGCG	3:329288	272
	GCACATCGGTAACGCG	3:329016	
*scpB*	CTGCAACGAACGACGCG	2:1062555	294
	GACGGAAGGGGAAGGGC	2:1062261	
*cepI*	GCCTGCAGGGCACAACGACGCCTATCATGC	2:2087932	267
	GAACGAAGGTCTGCATGGATG	2:2088199	
PBP	CGTCGGGAACGAGGCCC	2:2983704	313
	CGATGGGTTGGCGGTGGG	2:2983391	
*phuR*	CGTGTCGATGATCCGCG	2:2973940	404
	CACAGGTGGTCTCCC	2:2974344	
acyltransferase	CGATACACTGTGAGCCG	2:446287	336
	GTCCTTCAGCACGCCG	2:445951	
*zmpA*	CTCGAGGCTGGCCGGTACTG	3:478051	638
	GGATCCAGACTGAAGGCGGACG	3:478689	
MST005	GCACGCCCGCGTCAGGCG	1:366108	325
	CGCAAGCCACCACTACCCC		
MST011	CCTTGCTGAGATTGCCGGC	1:779005	321
	GACAGCGCGTTCACGGGCG		
MST028	CGTGTCGTTGCGGCGCGC	1:1484174	451
	GTCTGGCTGTACGCACGCC		
MST052	CCGTCATTTGTCGTCGGGC	1:3009328	341
	CCAGTCCATCGTGGCCGC		
MST059	CGCCTTCGGCAGCCCCG	1:3488873	315
	GCTGGTCGAGCAGCAGCGG		
MST068	CGTCGAGCGTCAGCTTGCGC	2:11203	325
	GGTCGAGCGTCCCGCGC		
MST072	GCATCCAGCAGGCGCGC	2:84846	398
	CCGACGGGACCGCAGCCC		
MST112	GCAGGTCGCCATGCCGGG	2:2156170	441
	ACCACGCGTACGCGGGC		

Mutagenic Oligonucleotides			

CepBx103	GCGTCTTTACGCGTCGACCCTGTAAGAGTTACC		*Sal*I
CepBx104	GTCTTTACGCCGTCATATGGTAAGAGTTACCAG		*Nde*I
CepBx105	CGCCGTCACCCCTGCAGAGTTACCAGTTACAGG		*PstI*
CepBx106	GCCGTCACCCTGCTGCAGTTACCAGTTACAGG		*Pst*I
CepBx107F	ACGCCGTCACCCTGTAACTAGTACCAGTTACAGGCTCCTC		*Spe*I
CepBx107R	GAGGAGCCTGTAACTGGTACTAGTTACAGGGTGACGGCGT		*Spe*I
CepBx108F	CCGTCACCCTGTAAGAGTCTAGAGTTACAGGCTCCTCGTGC		*Xba*I
CepBx108R	GCACGAGGAGCCTGTAACTCTAGACTCTTACAGGGTGACGG		*Xba*I
CepBx109F	CACCCTGTAAGAGTTACCGTCGACAGGCTCCTCGTGCCGCGC		*Sal*I
CepBx109R	GCGCGGCACGAGGAGCCTGTCGACGGTAACTCTTACAGGGTG		*Sal*I
CepBx110F	CCTGTAAGAG TTACCAGTTAAGATCTCCTC GTGCCGCGCG CTG		*Bgl*II
CepBx110R	CAGCGCGCGG CACGAGGAGATCTTAACTGG TAACTCTTAC AGG		*Bgl*II
CepBx111F	AGAGTTACCAGTTACAGGGATATCGTGCCGCGCGCTGTAATG		*Eco*RV
CepBx111R	CATTACAGCGCGCGGCACGATATCCCTGTAACTGGTAACTCT		*Eco*RV
CepBx112F	GTTACCAGTTACAGGCTCCGTACGCCGCGCGCTGTAATGCAC		*Bsi*WI
CepBx112R	GTGCATTACAGCGCGCGGCGTACGGAGCCTGTAACTGGTAAC		*Bsi*WI
CepBx113F	CCAGTTACAGGCTCCTCGTCGACCGCGCTGTAATGCACGC		*Sal*I
CepBx113R	GCGTGCATTACAGCGCGGTCGACGAGGAGCCTGTAACTGG		*Sal*I

^a ^Locations reported as chromosome:nucleotide
